# Early osseointegration of two in-house 3D-printed porous titanium implant designs: an *in vivo* sheep study

**DOI:** 10.3389/fsurg.2026.1818355

**Published:** 2026-05-25

**Authors:** Anna B. Borgognoni, Sarah S. Freund, Jørgen Baas, Michael M. Bendtsen, Jeppe S. Byskov, Bahram Ranjkesh, Jens R. Nyengaard, Ruben Pauwels, Thomas Baad-Hansen

**Affiliations:** 1 Department of Orthopaedic Oncology, Aarhus University Hospital, Aarhus, Denmark; 2 Additive Manufacturing, Danish Technological Institute, Aarhus, Denmark; 3 Department of Dentistry and Oral Health, Aarhus University, Aarhus, Denmark; 4 Core Center for Molecular Morphology, Department of Clinical Medicine, Aarhus University, Aarhus, Denmark; 5 Department of Pathology, Aarhus University Hospital, Aarhus, Denmark

**Keywords:** 3D printing, histomorphometry, osseointegration, porous surface design, push-out test, titanium implants

## Abstract

**Introduction:**

Early osseointegration is critical for long-term implant stability, and in-house 3D–printed porous surfaces may enhance bone integration by promoting cellular migration and tissue formation, provided that porosity is balanced with sufficient mechanical strength. This randomized animal study assessed whether statistically significant differences exist between custom 3D–printed titanium implants with a pore size of 630 μm and porosity of 73.9% (P1) and implants with a pore size of 690 μm and porosity of 69.1% (P2) regarding mechanical and histological osseointegration.

**Methods:**

Twenty stable, non-weight– bearing cylindrical implants (6 × 10 mm), comprising ten P1 and ten P2 implants, were randomly inserted into the medial left femoral condyle of 20 skeletally mature sheep. After 4 weeks, the animals were euthanized, and each specimen was divided into one block for mechanical axial push–out testing and another for histomorphometric evaluation.

**Results:**

Statistical analysis using the Mann–Whitney U test demonstrated no significant differences between P1 and P2 in strength (MPa; *p* = .87), energy (J/m2; *p* = .80), or stiffness (MPa/mm; *p* = .66). Histomorphometric analysis likewise revealed no significant differences in bone or fibrous tissue fractions between groups, and no foreign body reaction was observed in any specimen.

**Discussion:**

Within the limitations of this study, no statistically significant differences were detected between the two porous implant designs with respect to early osseointegration, and future research should investigate their performance in weight–bearing conditions.

## 1 Introduction

Osseointegration (OI) refers to the process by which tissue grows into or along the surface of an implant (1) and is achieved by ensuring rigid fixation by ingrowth of new bone (2). Aseptic loosening—mechanical instability without infection—is considered a leading cause of prosthesis failure (3). It has been showed that early bone ingrowth prevents formation of a thick fibrous membrane at the interface which may be responsible for preventing a solid fixation in the long term (4). The insufficient OI or lack of initial stability of the implant is regarded as a primary mechanism of aseptic loosening (2). Consequently, it is essential that implant design and fabrication prioritize factors that promote optimal bone fixation. Implants with porous surfaces enhance the amount of OI by increasing permeability and facilitating cellular migration onto the implant surface (5). Pores provide additional space for cellular interactions and new tissue formation, thereby promoting favourable biological responses (6). However, increased porosity can compromise the mechanical integrity of the scaffold, necessitating a balanced design approach to optimize both biological and mechanical properties (7). As a result, ongoing investigations aim to further refine implant designs (8).Traditionally, porosity in metal implants has been achieved through coating techniques involving powder, beads, or fibers (9). In contrast, 3D printing offers superior control over pore size, shape, and distribution, which allows for the fabrication of intricate internal lattice structures that mimic the mechanical behaviour of natural bone, potentially leading to improved long-term stability and reduced complication rates (10). The integration of emerging technologies, however, presents significant challenges, primarily due to limited published preclinical data on new commercial implants and the lengthy development timelines associated with modelling and manufacturing processes. Our institution benefits from an in-house 3D printing center, which enables the design and fabrication of patient-specific models and instruments (11, 12). Additionally, collaboration with the Danish Technological Institute (DTI) provides access to metal additive manufacturing facilities and engineering expertise. This study compares the early OI of two in-house custom-designed implants in an *in vivo* model. The two implant designs were selected to investigate whether modest variations in pore size and porosity within a clinically relevant range would influence early osseointegration. Implant 1 was characterized by a smaller pore size and higher porosity, whereas Implant 2 featured a larger pore size and lower porosity, while both designs maintained sufficient structural integrity for implantation.

## 2 Materials and methods

### 2.1 Study design

The study was designed as unpaired comparison with two groups. Twenty female Romney-Texel sheep with a mean weight of 65.5 kg (58–75.6 kg) and a mean age of 41.5 months (36–50 months) were included in the study. The study was approved by the Danish Animal Research Inspectorate (ID: 2022-15-0201-01240), conformed to Danish law and followed the ARRIVE 2.0 guidelines (13). The animals had not previously been subjected to experiments. Acclimatization lasted a week before surgery. The sheep were accustomed to roaming outdoors as they pleased during the observation period. The animals were cared for pre- and post-operatively with daily supervision in the experimental animal stable by trained animal assistants under the supervision of a veterinarian. During the first 3 days post-operatively, the sheep were observed three times daily. The staff were accustomed to observing sheep with this type of implant from previous experiments. Randomization was performed with a randomization program and reported in excel spreadsheet.

### 2.2 Sample size

A sample size calculation for unpaired randomized studies was done. The sample size was determined through *a priori* statistical power calculation, based on outcome variability (mechanical and histomorphometrical data) reported in previous studies using similar implant models and experimental designs (14–18). The selected sample size (*n* = 20 animals, 10 implants per group) was chosen to achieve 80% statistical power at a significance level of *α* = 0.05**.**

### 2.3 Implant design and production

After testing various porosities in terms of mechanical strength, two implants' designs (P1 & P2) were selected for this study. Implants were designed using 3-Matic 17.0 (Materialise, Leuven, Belgium) using a gyroid architecture. The selected designs were chosen following preliminary mechanical screening to identify structures within a clinically relevant porosity range while maintaining sufficient structural integrity. The chosen implants had pore sizes (P1: 630 µm; P2: 690 µm) and porosities (P1 73.9% P2: 69.1%) within the range of other works in recent literature (19, 20). Additional data can be found in Table 1.

**Table 1:** Implant characteristics.

Name	Topology wall thickness (mm)	Cell size (mm)	Min. channel diameter (mm)	Wall thickness (mm)	Porosity (%)
P1	0.3	2.2	0.63	0.22	73.9
P2	0.4	2.4	0.69	0.29	69.1

Data is shown as mean.

### 2.4 Implant design and production

**Figure 1:**
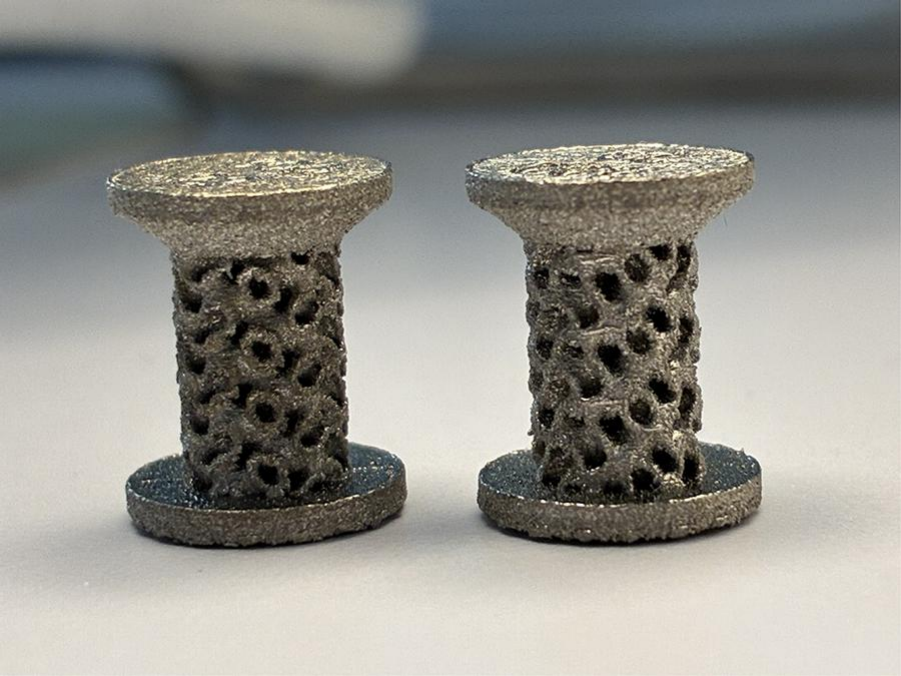
Gap-models utilized. On the left, an example of P1 implant; on the right, an example of P2 implant.

Implants were produced at the DTI, Aarhus, Denmark, using an SLM machine (SLM® Twin, SLM Solutions, Lübeck, Germany) with a twin 400W IPG fibre laser with a layer thickness of 60 µm. The Ti6Al4V-grade 23 ELI powder (SLM Solutions, Lübeck, Germany) used had a particle size between 20 and 63 µm and a mass density of approximately 4.43 g/cm^3^. The implants were cylindrical, measuring 10 mm in height and 6 mm in diameter with a footplate of 10 mm in diameter and 1 mm in height on both ends of the implant. When inserted into a 10 mm drill hole, this centred the implant and provided a uniform 2-mm gap around it (Figure 1).

All implants were sterilised using an autoclave.

### 2.5 Anesthesia, pain management and handling of animals

Pre- and postoperatively, sheep received intramuscular Noromox (150 mg/mL) at a dosage of 15 mg/kg as antibiotic prophylaxis. Metacam was administered for analgesia both before and after surgery. Additionally, a fentanyl transdermal patch (75 µg/h) was applied for 3 days as postoperative pain management. All animals were permitted unrestricted weight-bearing.

### 2.6 Surgery

Under general anesthesia and aseptic conditions, a 5 cm skin incision was made at the distal femur using electrocautery. The vastus medialis muscle was detached at the articular capsule to expose the metaphyseal bone. A guide wire was centrally inserted proximal to the cartilage, perpendicular to the bone surface. Using a cannulated drill (Ø 10 mm), a cylindrical cavity measuring 12 mm in depth was created over each guide wire. Excess periosteum at the edge of the defect was removed with a scalpel, and loose bone fragments were cleared. Each implant was placed using an impaction tool to ensure consistent central positioning. All 20 implants were placed by a single surgeon. Following a 4-week observation period, the sheep were sedated and euthanized with a hypersaturated barbiturate overdose.

### 2.7 Specimen preparation

**Figure 2:**
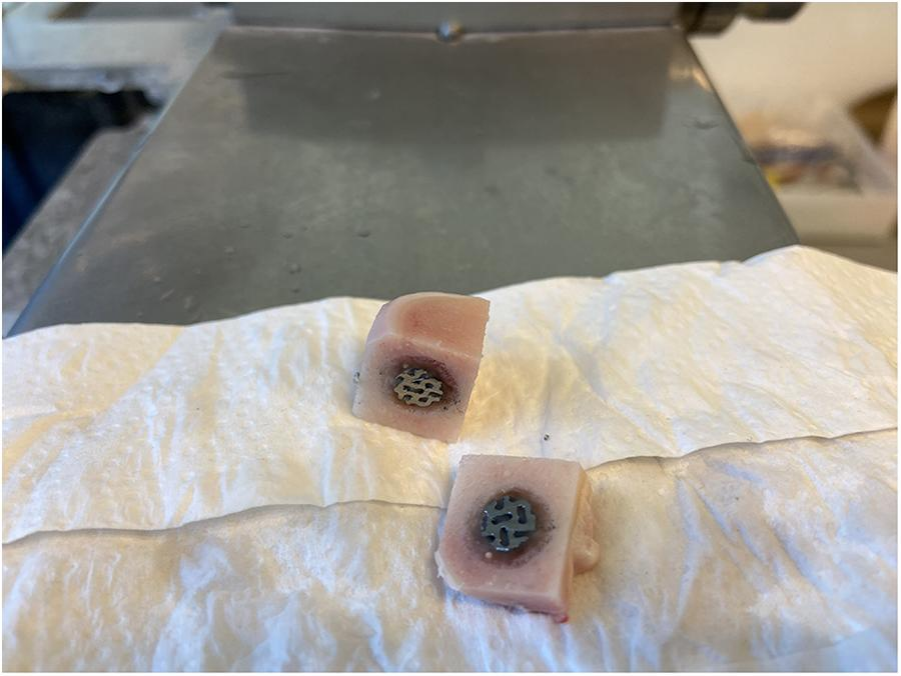
Sample preparation. At retrieval, an approximately cubic piece of ca. 2 cm × 2 cm × 2 cm was obtained for the following analyses and divided in 2 blocks (3 and 6 mm).

Two sections were obtained from the medial femur, perpendicular to the implant axis with a water-cooled diamond band saw (Exact Apparatebau, Nordenstedt, Germany), as shown in Figure 2. A 3-mm thick outer section was stored at −20 °C for mechanical testing, while the inner section was processed for histomorphometry: it was dehydrated in graded ethanol (70%–100%), embedded in methylmethacrylate (Technovit 7200 VCL; Exact Apparatbau, Nordenstedt, Germany), and cut into four 50-μm vertical sections from the central part of the implant using a microtome (KDG-95, MeProTech, Heerhugowaard, The Netherlands). These sections were stained with toluidine blue and mounted on glass slides. Woven and lamellar bone were identified by their morphological features.

### 2.8 Biomechanical testing

**Figure 3:**
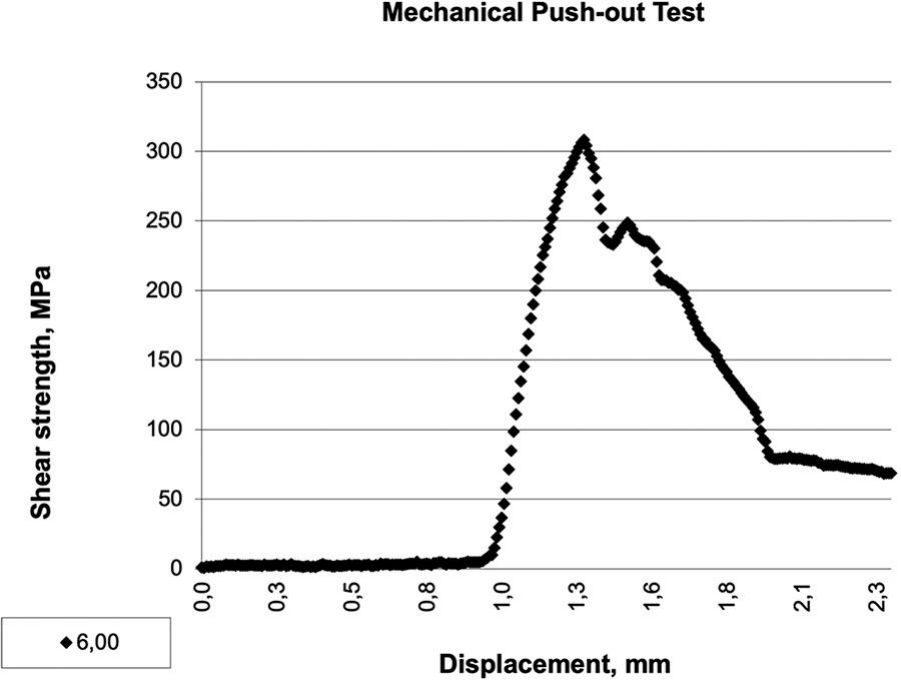
Mechanical tests.

Specimens underwent axial push-out testing using an Instron Universal test machine (Instron Ltd., High Wycombe, UK). Positioned cortical side up on a support jig with a 7.4 mm opening, implants were pressed out at 5 mm/min following a 0.5 N preload. Load and displacement were continuously recorded to calculate ultimate shear strength (the maximum load the connection can withstand normalized by an approximation of the surface area), apparent shear stiffness (a measure of a shear connector's resistance to deformation, calculated from the force-slip or force-displacement data recorded during the test), and total energy absorption (how much energy a material or composite can absorb when a compressive force is applied to push it out or deform it.). Ultimate shear strength was calculated as maximum force normalized to the estimated implant–bone interface area. Apparent shear stiffness was derived from the slope of the linear elastic portion of the load-displacement curve, while total energy absorption was calculated as the area under the load-displacement curve until implant displacement. Figure 3 shows an example of the obtained data curve.

### 2.9 Histomorphometry

Four central sections, each 50 µm thick, were cut parallel to the implant axis via the Vertical Uniform Random (VUR) sectioning method from the central part of the implants using a hard-tissue microtome. This approach offers highly reliable results with minimal bias (13, 15). Regions of interest (ROIs) were defined using the open-access stereological software FIJI (21) and a semi-automated macro script:

- ROI 1: A midline was delineated at the outermost edges of the implant surface, then a surface zone was defined from −500 μm extending into the implant and +500 μm into the 2 mm gap.
- ROI 2: a gap zone, spanning from the end of ROI 1 and ranging 500–1,500 μm into the gap.
- ROI 0: implant body.

**Figure 4:**
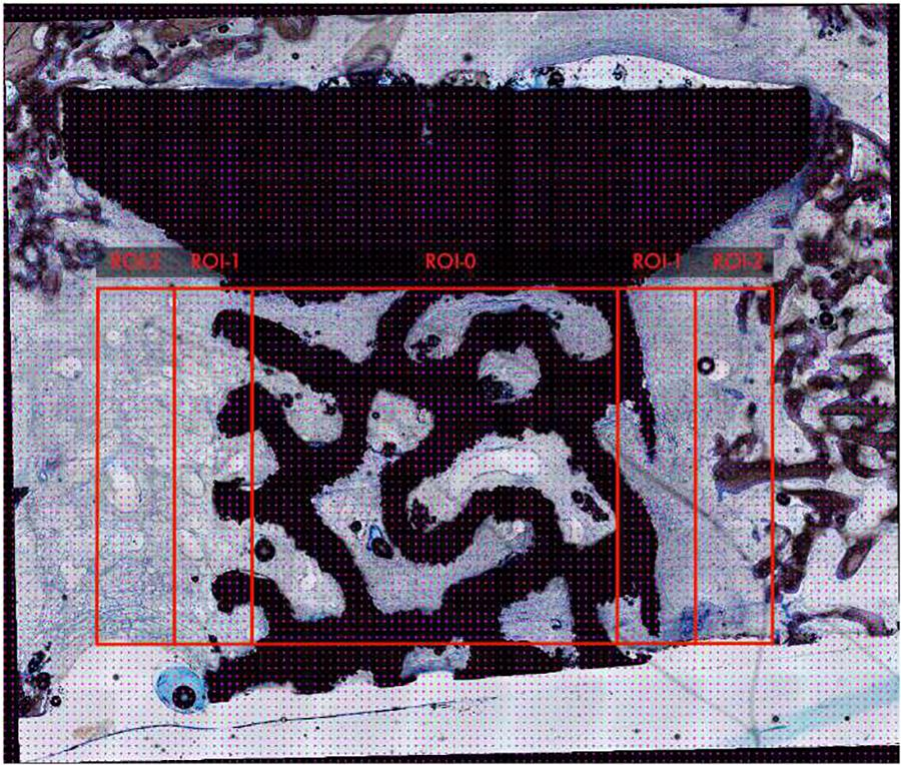
An example of the point-counting technique. For every point in the ROI the corresponding tissue is noted.

Blinded quantitative histomorphometry was carried out with FIJI and newCAST (Visiopharm A/S). The Cavalieri method was employed. Point counting was used to estimate the area of various ROIs: a grid is randomly placed over the ROI, and points that hit the ROI are counted. Both the random placement of grids and systematic sampling of tissue sections are crucial for unbiased results. In ROI 2, the sampling fraction was set at 100% (the whole region was observed), and volume fractions of new bone, loose fibrous tissue, and foreign body membranes were estimated. A mean of 832 points was counted in this zone for every slide. For ROI 1, a line-intersection technique was used to estimate bone growth directly on the implant surface, which was considered more suitable for this purpose (22). For this technique, the number of intersections counted was intended to provide highly reproducible estimates of tissue fractions above 10%. The reliability of a tissue fraction estimate is directly related to the number of line intersection counts on which the estimate is based. In accordance with stereological principles, a tissue fraction estimate should be based on 100 counts, however if the differences between the tissue is large fewer counts can be acceptable. In our sample, bone tissue in contact with the surface was scarce, so around 50 counts were deemed enough. Here a mean of 251-line intersections/slide were counted. An example is shown in Figure 4.

ROI 0 was not analysed, as the bone in this zone was extremely scarce.

### 2.10 Statistical analysis

All statistical analyses were made using R (v4.3.1; R Core Team 2021). The datasets from mechanical and histological tests were tested for normality with the Shapiro–Wilk test. The data were not normally distributed and were evaluated with the Mann–Whitney *U* test. For all comparisons, the significance level was set at 0.05.

## 3 Results

All 20 sheep were fully weight-bearing within 2 days after surgery and completed the 4-week observation period without signs of distress, or other procedure-related complications. Only one sheep showed signs of superficial infection and was treated with antibiotics. No implants showed signs of infection at harvest.

Two implants (one in each group) did not withstand the initial 0.5 N preload required to define contact position and were therefore excluded from biomechanical analysis only. Histomorphometric analyses were performed on all specimens. Final sample sizes were therefore *n* = 9 per group for biomechanical testing and *n* = 10 per group for histological evaluation.

**Figure 5:**
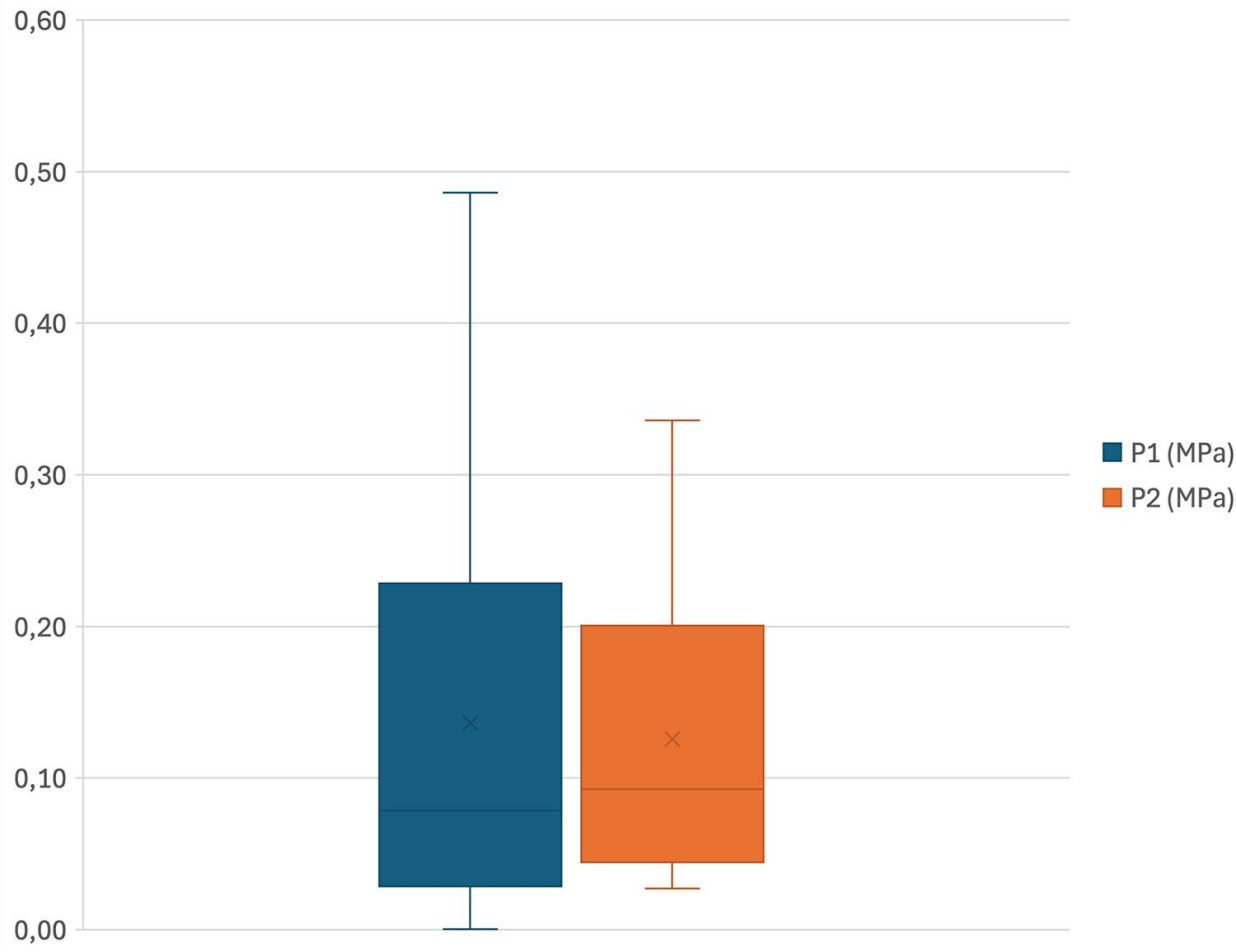
Boxplot showing ultimate shear strength results (MPa).

**Figure 6:**
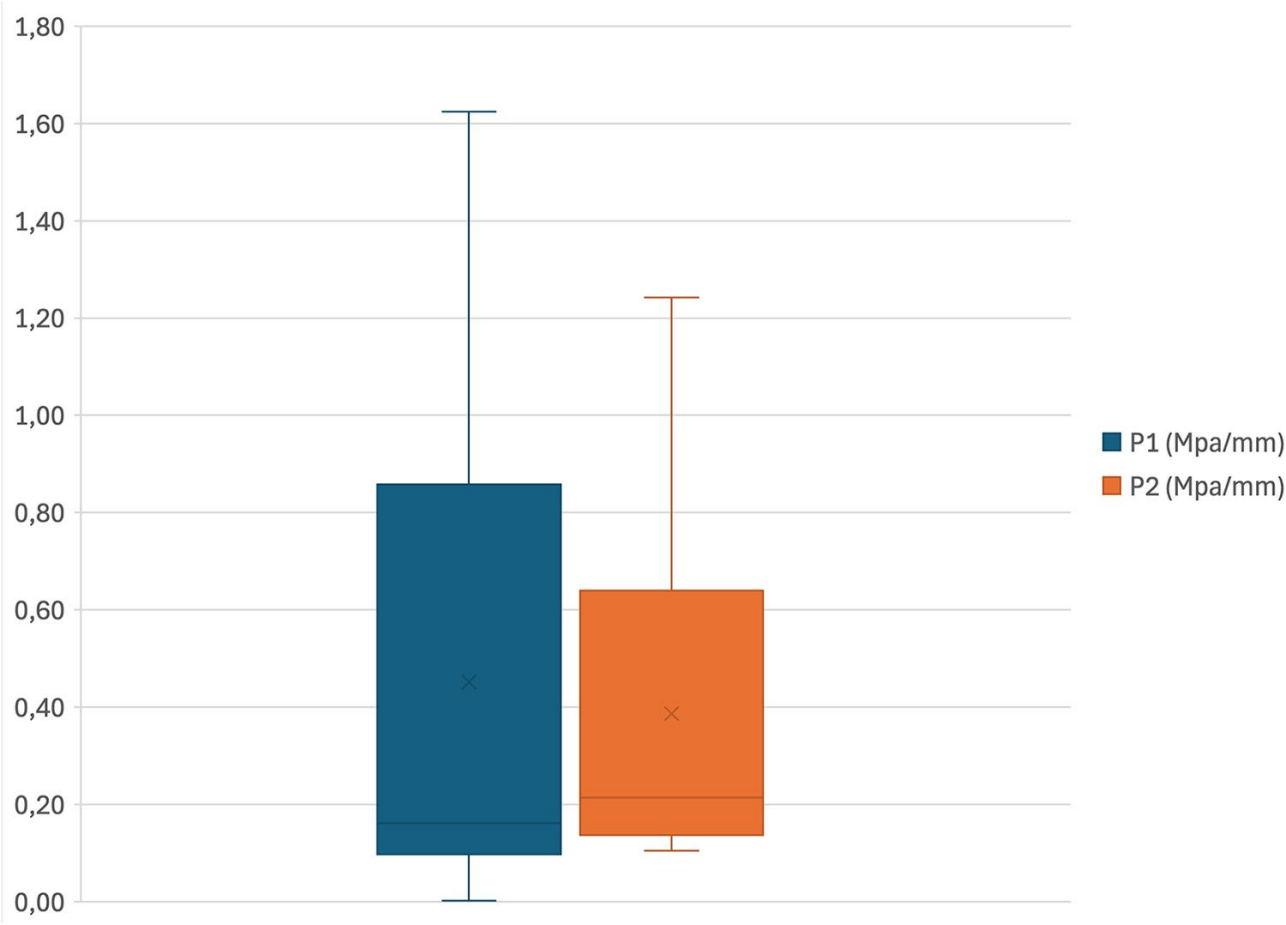
Boxplot a showing apparent shear stiffness results (MPa/mm).

**Figure 7:**
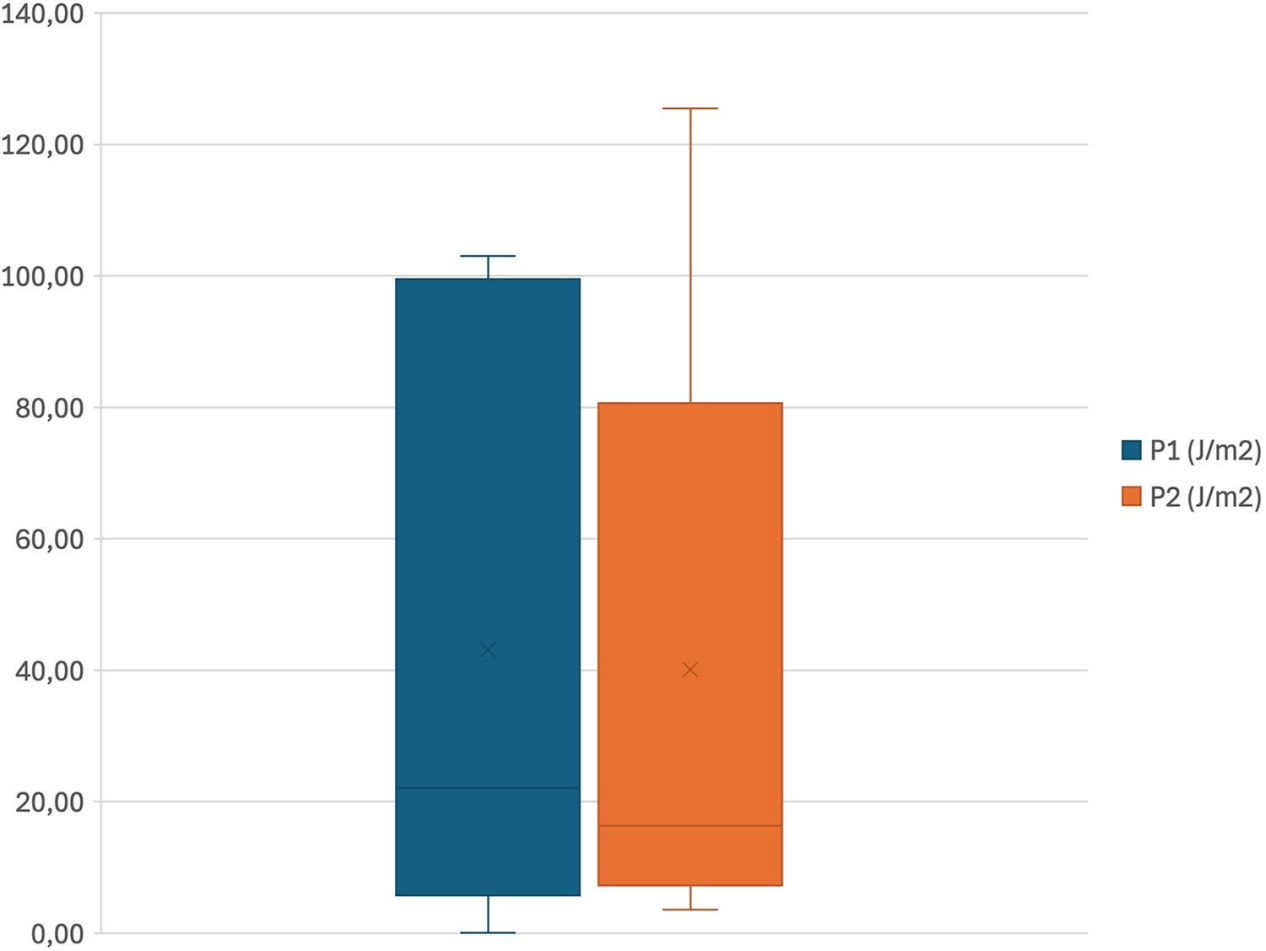
Boxplot showing total energy absorption results (J/m^2^).

Mechanical testing revealed no statistical difference between the two implants, in terms of strength, energy and stiffness. Results are presented in Figures 5–7.

Histomorphometrical analysis similarly showed no significant difference between the two groups, and no foreign body reaction was detected in any of the implants. In ROI 2, the percentages of bone and loose fibrous tissue were similar in both groups (Table 2). A small percentage of tissue was classified as artifacts and excluded from the results. In ROI 1, results were also statistically non-significant for bone Sv and loose fibrous tissue Sv (Table 3 μm^−1^).

**Table 2:** Histomorphometric ingrowth in gap zone (ROI 2), point-counting technique.

Implant	Bone (fraction)	Fibrous (fraction)
P1	0.16 (0.08)	0.63 (0.93)
P2	0.15 (0.10)	0.61 (0.10)
Mann–Whitney *U* test	*p* = 0.97	*p* = 0.88

**Table 3:** Implant surface ongrowth (ROI 1), line-intersection technique.

Implant	Bone (*S_v_*)	Fibrous (*S_v_*)
P1	0.01 (0.01)	1.72 (0.24)
P2	0.01 (0.03)	1.65 (0.20)
Mann–Whitney *U* test	*p* = 0.91	*p* = 0.39

*S_v_* [median (interquartile range)] in µm^−1^.

## 4 Discussion

The present study evaluated the early osseointegration of two in-house designed and additively manufactured porous Ti6Al4V implants differing modestly in pore size and porosity. Mechanical push-out testing and histomorphometric analysis demonstrated no statistically significant differences between the two implant designs after 4 weeks of implantation in a sheep femoral condyle model. These findings suggest that within the tested parameter range, the differences in pore architecture were insufficient to produce detectable differences in early OI under the present experimental conditions.

The influence of pore size and porosity on OI has been extensively investigated, with previous studies suggesting that both parameters strongly affect bone ingrowth, vascularization, and mechanical fixation. Taniguchi et al. (23) reported significantly improved bone ingrowth in porous titanium implants with a pore size of 600 µm compared with implants featuring pore sizes of 300 and 900 µm in a rabbit model, indicating that intermediate pore sizes may optimize early fixation. Similarly, Ragone et al. (24) demonstrated extensive OI of additively manufactured trabecular titanium implants in sheep after an 8-week observation period, while Crovace et al. (25) reported successful long-term integration of highly porous titanium scaffolds in large critical-sized bone defects after 12 months. Collectively, these studies suggest that pore architecture plays a critical role in implant integration. In general, a larger pore size facilitates neovascularization but may compromise mechanical integrity and diminish cell adhesion. Conversely, smaller pore sizes enhance both cell adhesion and tissue formation, although they also increase the risk of pore occlusion (26).

A comparison of outcomes across studies investigating metallic implants remain challenging. In a review from Gu et al. (27) most of the included studies fail to report sample size calculation. Only 63% of the reported studies in the review mentioned the type of powder used for implant production and only 65% of the studies mention the post-processing applied. In the above-mentioned review, the authors recommend following the International Organization for Standardization ((ISO10993-6:2016(E)) when planning an *in-vivo* study, both regarding animal models, dimensions of implants and follow-up time coherent with the aim of the study. The characteristics of this study and implant design were therefore chosen according to these guidelines: adult sheep were selected for the similarity with human in weight, metabolism, and bone-remodelling rates. Recommended implants size for large animal models was 4 × 12 mm and recommended short-term outcome as early OI was 1–4 weeks. The aforementioned systematic review showed that most studies using Ti-6Al-4V scaffolds have gravitated toward implants with pore sizes ranging from 300 to 800 µm and a porosity between 55% and 78% (5). Gu et al. (27) suggested that the ideal porosity should be around 80%–90%, even though most of the studies choose a lower porosity of 60%–70% to ensure optimal mechanical strength. Both of our implants had pore sizes (P1: 630 µm; P2: 690 µm) and porosities (P1 73.85% P2: 69.06%) falling within this optimal range.

The absence of significant differences in the present study likely reflects the aforementioned experimental factors. First, the differences between the two implant designs were relatively modest. Both designs fall within ranges reported to support favorable bone ingrowth, and it is therefore possible that both structures were already positioned within an effective biological window for early OI. Consequently, the architectural differences may not have been sufficiently large to induce measurable biological differences.

Second, the 2 mm unloaded gap model likely limited direct interaction between newly formed bone and the implant surface during the early healing phase. Histological analysis confirmed that the peri-implant gap was predominantly occupied by loose fibrous tissue, with only limited bone formation at 4 weeks. This observation suggests that the model primarily captured the earliest stages of tissue response, before substantial bone bridging and mature OI could occur. Under such conditions, subtle effects of pore architecture may be masked.

This interpretation is supported by previous work using unloaded gap models, which consistently demonstrates reduced early fixation compared with press-fit designs. Søballe et al. (9) and Daugaard et al. (18) showed that increasing implant-bone distance substantially impairs early mechanical fixation and delays bone formation. While the unloaded gap model provides a clinically relevant simulation of compromised bone stock, particularly relevant for oncologic reconstruction where large peri-implant gaps frequently occur, it may reduce sensitivity for detecting differences attributable solely to surface architecture.

The relatively short 4-week observation period represents another important limitation. Early OI is characterized by inflammatory regulation, fibrous tissue organization, initial woven bone formation, and subsequent remodeling. Several studies have shown that architectural differences often become more evident at later healing stages, when mature bone infiltration and remodeling are more advanced (24, 25, 27). The short follow-up in the present study was intentionally selected to assess early biological performance but may have limited our ability to detect delayed architectural effects.

Despite the lack of measurable differences between the two implant designs, several findings remain clinically relevant. Both implant configurations were successfully designed, manufactured, sterilized, and implanted without technical complications. The implants were well tolerated by all animals, no foreign body reactions were observed, and postoperative recovery was rapid. These findings support the feasibility of producing custom porous titanium implants through an in-house development pipeline. This feasibility is particularly relevant for orthopedic oncology and complex revision surgery, where patient-specific implants are often required to address irregular bone geometry and compromised bone stock.

The present study also highlights the complexity of optimizing porous implant design. Pore size, porosity, topology, wall thickness, and interconnectivity are highly interdependent variables. Modifying one parameter inevitably influences others, making it difficult to isolate the biological effect of any single structural characteristic. This interdependence underscores the importance of systematic experimental testing when developing clinically applicable porous implants.

Future studies should evaluate implant performance under conditions more sensitive to architectural variation, such as press-fit implantation, reduced gap sizes, or longer observation periods. Additional investigation under load-bearing conditions would further improve clinical relevance and help determine whether design-related differences emerge during functional mechanical adaptation.

Within the limitations of this study, our findings indicate that both porous designs demonstrate comparable early biological performance in an unloaded large animal gap model. While no statistically significant differences were detected, the successful implementation of both designs supports the continued development of in-house designed porous titanium implants for future patient-specific orthopedic applications.

## 5 Study limitations and future perspective

This study has limitations: the study design is unpaired and the gap model is unloaded. It was also observed that, despite a gap of 2 mm being well within the range found in the literature, it played a major role in OI when compared to other surface characteristics such as pore density and pore size. The absence of detectable differences between groups should be interpreted in light of the experimental model. The relatively large 2 mm gap and short observation period likely limited direct bone–implant interaction, thereby reducing sensitivity to detect differences attributable to pore architecture. To better observe the influence of these parameters on early OI, future studies will need to test lower gaps or press-fit models or alternatively observe how porosity influences late OI. While the gap-model has a lower OI than the press-fit model, it allows to observe the deposition of new tissues within the gap and around the implants. This will allow future studies to observe the influence of other treatments on the periprosthetic bone growth and the effects on the surface new-bone/prosthesis.

## 6 Conclusion

In line with our aims, 20 custom-made 3D-printed metallic implants were designed, produced, implanted, and studied in a large-animal models. It was observed that the model design and implant production were accomplished quickly and efficiently, and the implants produced were well tolerated, with no signs of foreign body reaction. Within the limitations of this study, no statistically significant differences were detected between the two porous implant designs with respect to early osseointegration. While future research is still needed to strengthen designs and production pipelines, this work is a first step not only to show that implant production in-house is feasible, but to serve as a first step for the future design and production of custom patient-specific tailored-made 3D printed metallic protheses.

## Data Availability

The raw data supporting the conclusions of this article will be made available by the authors, without undue reservation.
